# Supplementary figures and images for: Characterization of the complete chloroplast genomes of five Populus species from the western Sichuan plateau, southwest China: comparative and phylogenetic analyses
Source: PeerJ. 2019 Feb 20;7:e6386. doi: 10.7717/peerj.6386 (PMC6387583; doi:10.7717/peerj.6386)

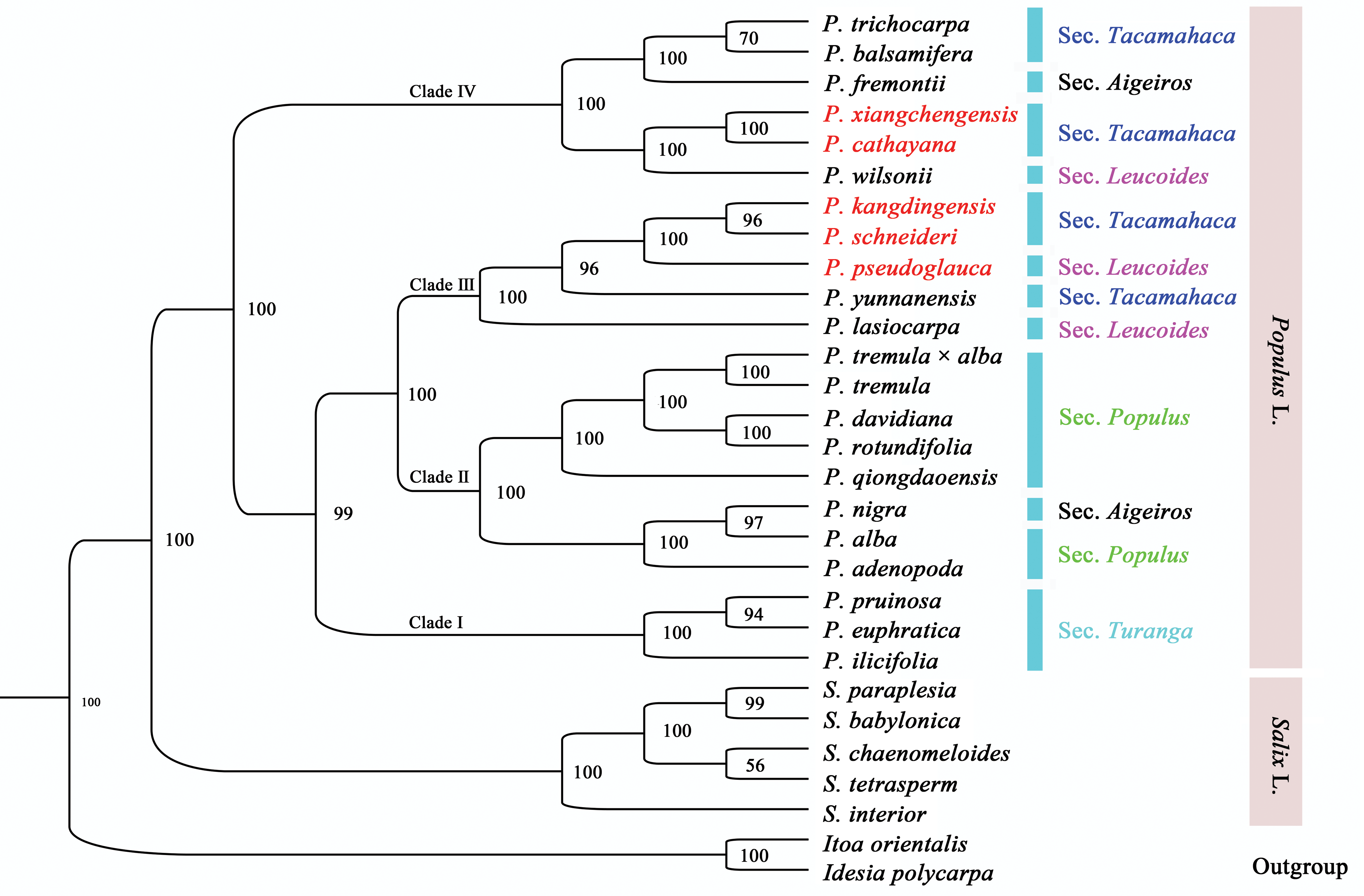

Supplement: Supplemental Information 8 — The numbers associated with each node are bootstrap support values. [file peerj-07-6386-s008.png]

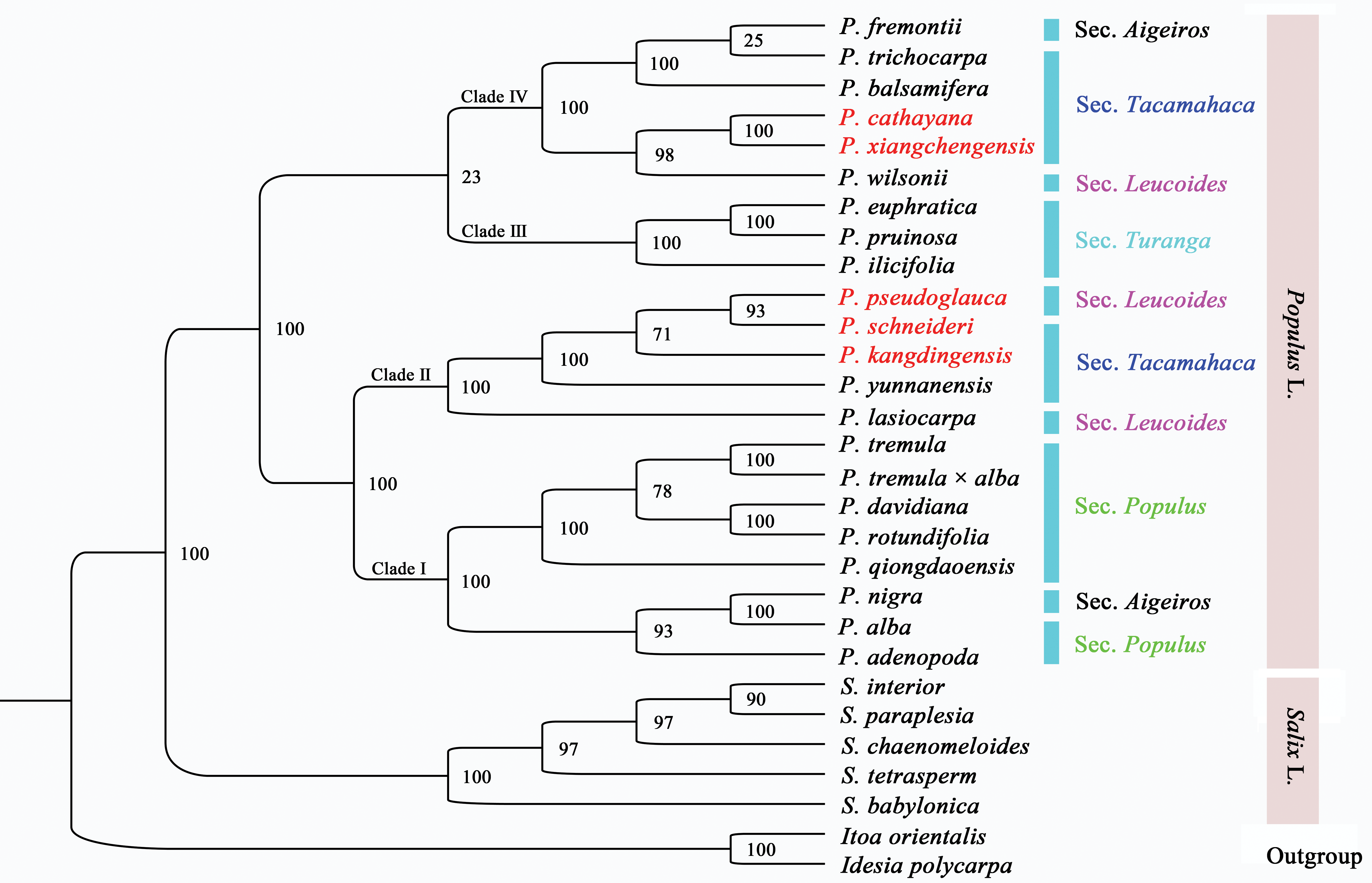

Supplement: Supplemental Information 9 — The numbers associated with each node are bootstrap support values. [file peerj-07-6386-s009.png]

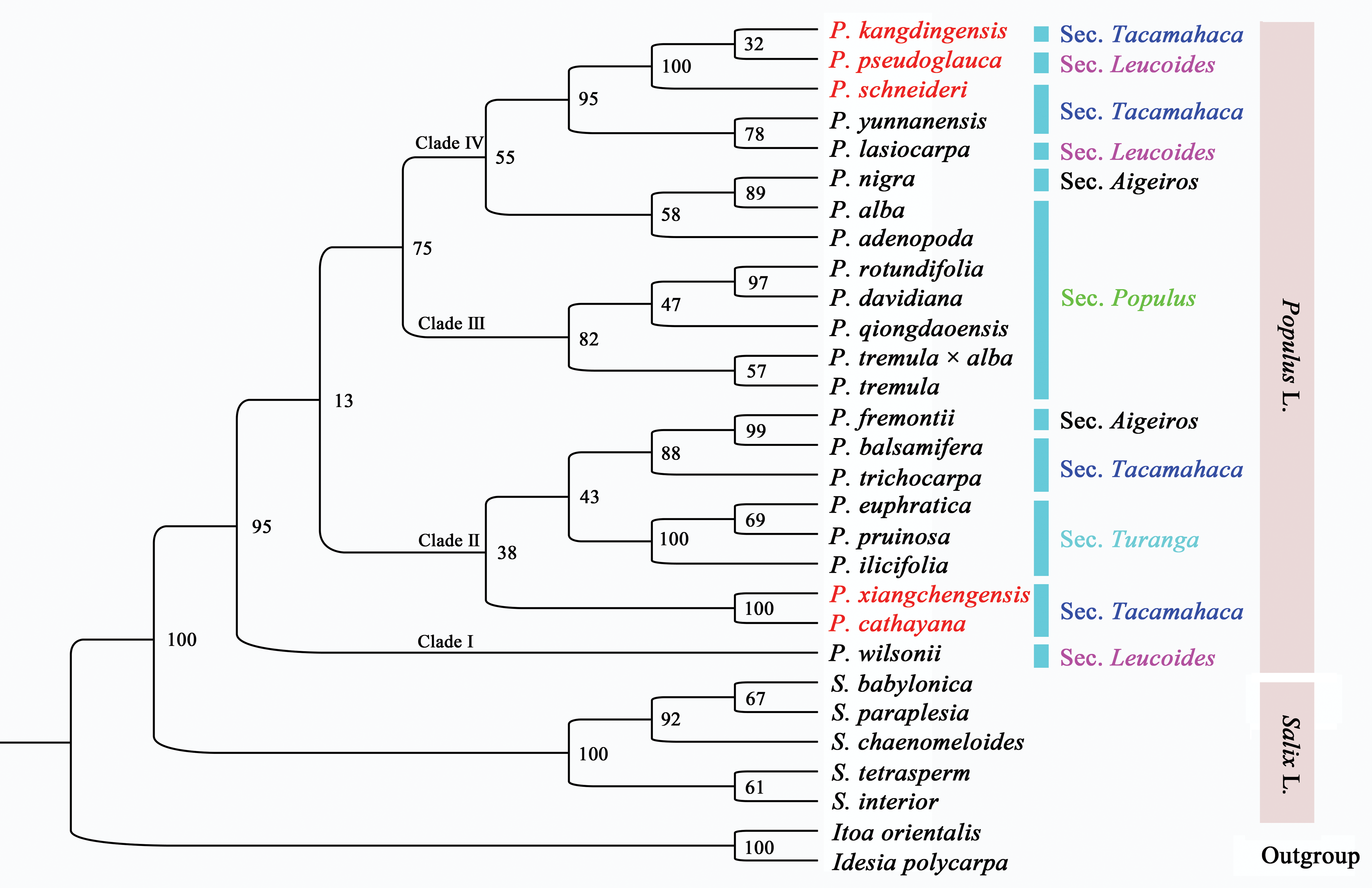

Supplement: Supplemental Information 10 — The numbers associated with each node are bootstrap support values. [file peerj-07-6386-s010.png]

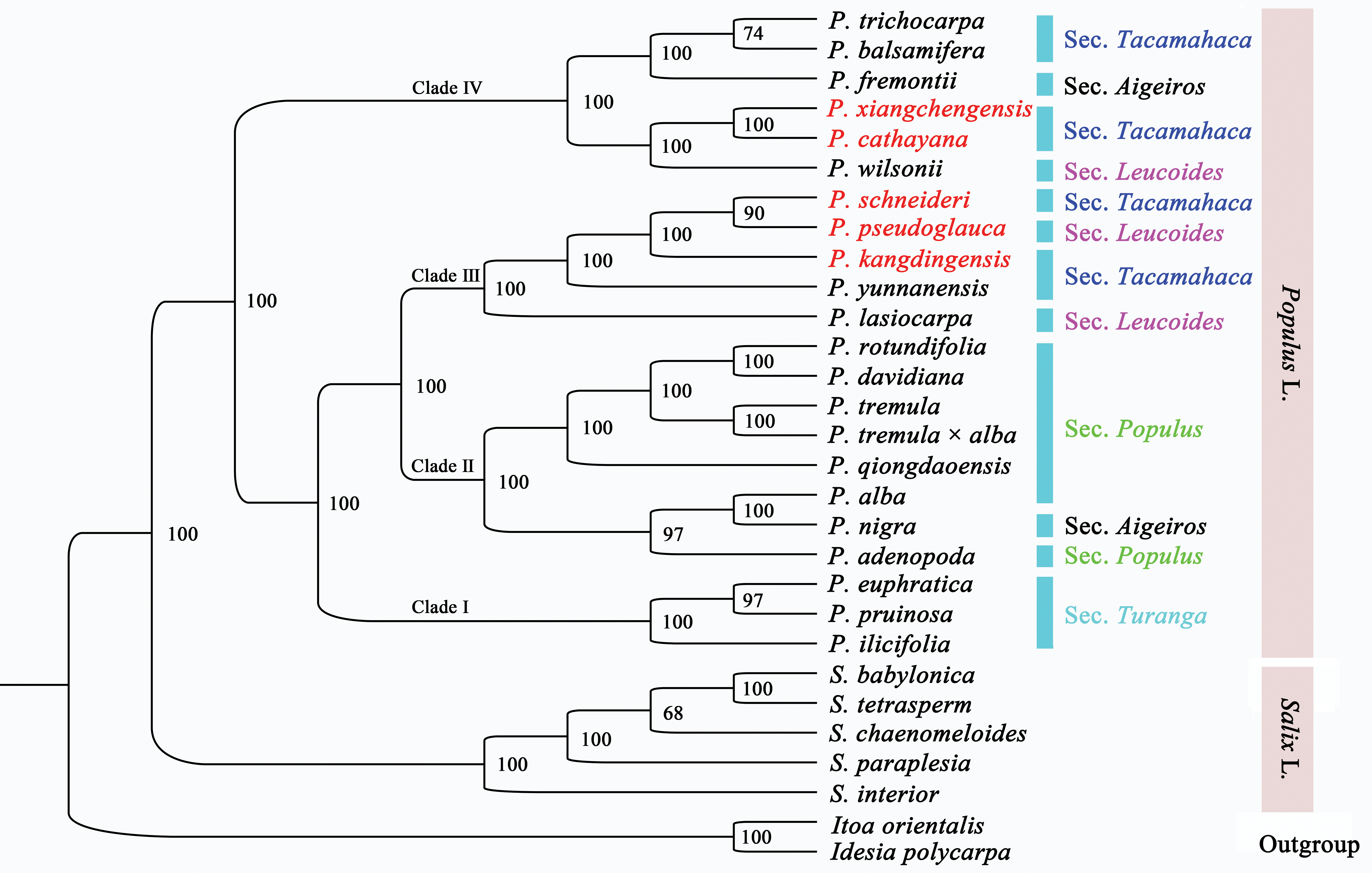

Supplement: Supplemental Information 11 — The numbers associated with each node are bootstrap support values. [file peerj-07-6386-s011.png]

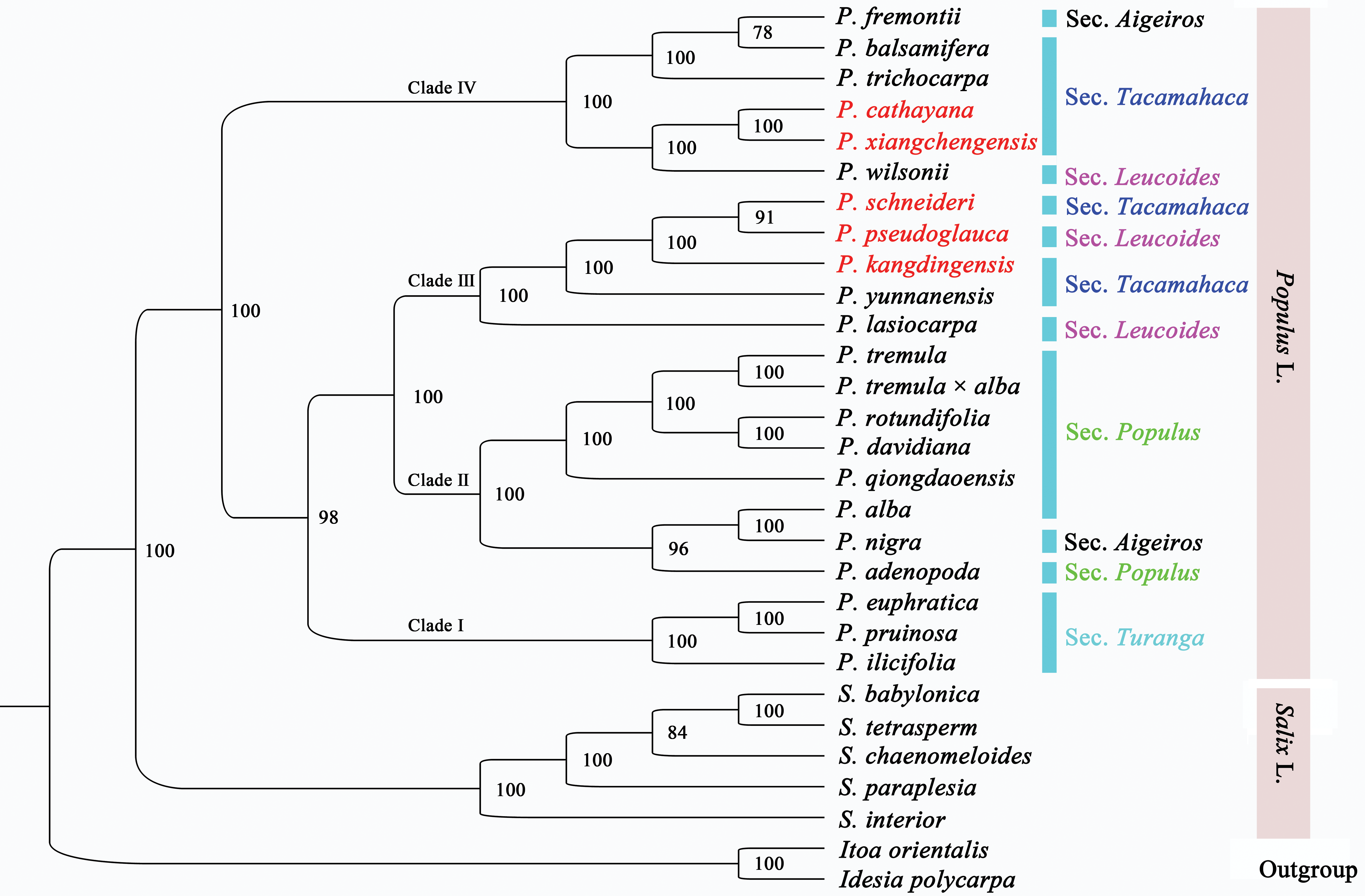

Supplement: Supplemental Information 12 — The numbers associated with each node are bootstrap support values. [file peerj-07-6386-s012.png]

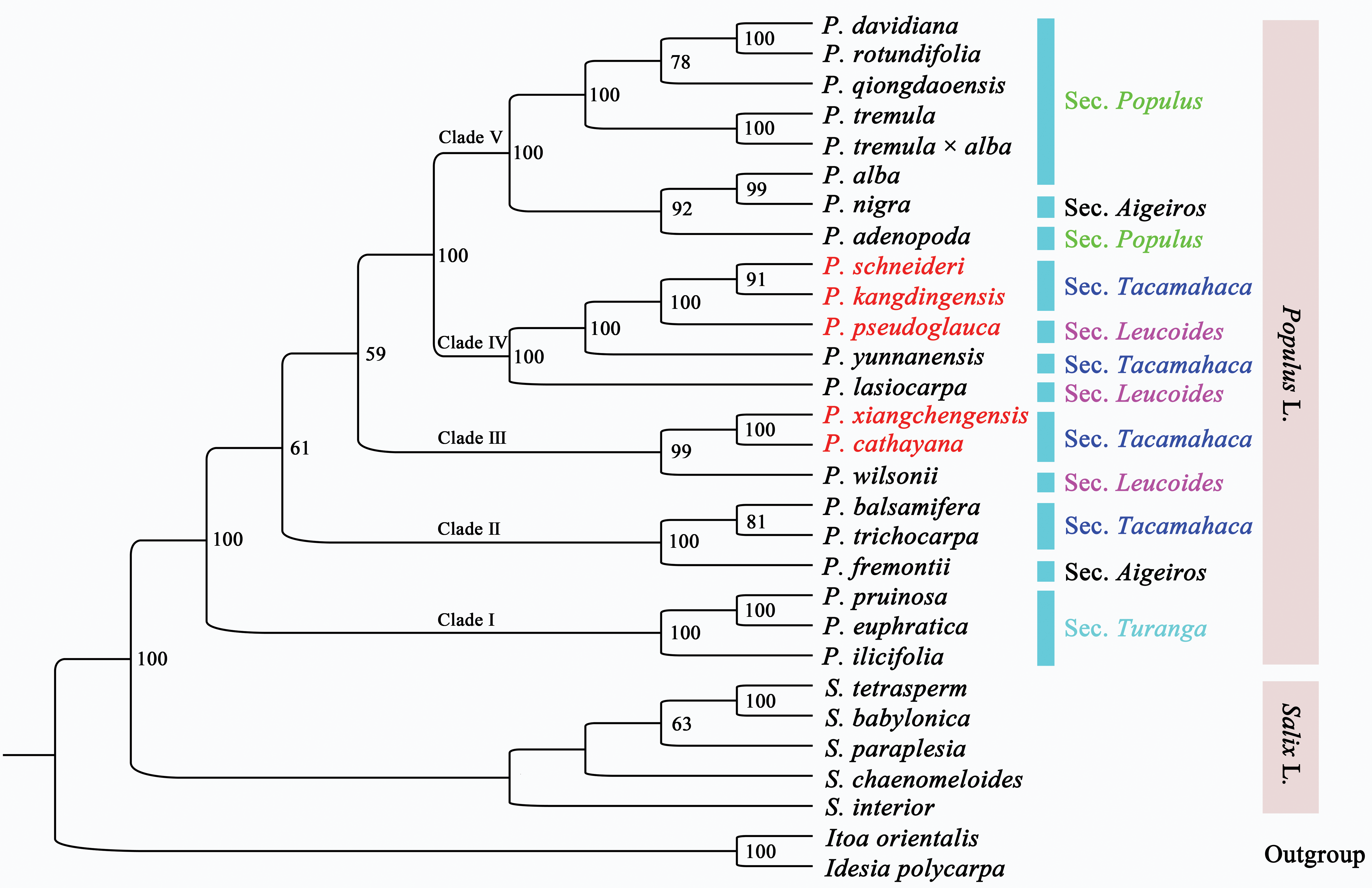

Supplement: Supplemental Information 13 — The numbers associated with each node are bootstrap support values. [file peerj-07-6386-s013.png]
